# The use of 5‐fluorouracil in the prevention of tendon adhesions: A systematic review

**DOI:** 10.1002/ame2.12110

**Published:** 2020-04-03

**Authors:** Omid Nazifi, Andrew L. Stuart, Dariush Nikkhah

**Affiliations:** ^1^ Royal Perth Hospital Perth WA Australia; ^2^ Locum Consultant Plastic Surgeon Department of Plastic and Reconstructive Surgery Royal Free London NHS Foundation Trust London UK

**Keywords:** 5FU, animal models, physiology, plastics, tendon

## Abstract

**Background:**

This systematic review aims to study the effectiveness of 5‐fluorouracil (5FU) in the reduction of tendon adhesions postsurgical repair.

**Method:**

A systematic review was performed involving four databases, Cochrane, PubMed, MEDLINE, and Embase, looking for evidence of at least Level I or Level II (according to NHMRC) in the use of 5FU in tendon repairs in human or animal studies.

**Results:**

Across the four databases 546 articles were identified. Of these 12 were identified as relevant, with a further two being excluded after in depth review.

**Conclusions:**

Nine of the 10 studies showed that 5FU was effective at reducing tendon adhesions in their respective animal studies. One of the 10 studies showed no significant change compared to the control group.

## 1 INTRODUCTION

The burden of tendon injuries places a tangible strain on the patient, the economy, and the hospital system. It is a significant source of socioeconomic stress and can have a pronounced effect on the daily life of those who suffer these injuries.^1^ After the initial injury, tendon adhesions often further hamper recovery, with the injured tendon often permanently losing strength and functionality.^2^ Despite this, a treatment that reliably, safely and effectively treats tendon adhesions still remains elusive.

Research into this area has produced many suggestions as to how this problem is best solved, recognizing that good surgical technique must go hand in hand with the modulation of the healing response.^3^ In this regard, 5‐fluorouracil has been identified as a drug that is able to modulate the immediate inflammatory response, from only a single application with no systemic side effects.^4^ Indeed its use in ophthalmic surgery has similarly shown to produce a long‐term reduction in the presence of scarring after glaucoma surgery.^5^, ^6^, ^7^

It is with this in mind that the authors have undertaken a systematic review of the current literature to assess the effectiveness of the use of topical 5FU in a “single touch” technique on the reduction of adhesion formation in repaired tendons.

## 2 METHODS

This systematic review aims to investigate the following population, intervention, control, and outcome question: in humans, cadaveric, and animal models, undergoing tendon repairs, compared to control techniques, what is the efficacy of the use of 5FU in preventing postoperative tendon adhesions?

To address this, we performed a systematic review of the available medical literature using several medically relevant databases, including Cochrane, PubMed, MEDLINE, and Embase.

Two authors independently performed the search on the 22 April 2019. Database journal search dates ranged from 1950 to the current. A range of search items were used to gain all articles related to the topic. Search terms included a variety of combinations including tendon adhesions, tendon repair, cytotoxic agents, and fluorouracil. Several lines of level I and II evidence (according to the NHMRC) were used in the review. Potential inclusive papers were manually screened by the authors, discussed, and a decision made regarding inclusion or exclusion. The full manuscripts of the remaining articles were reviewed and reference lists checked for potential studies not identified by our original search. Authors from these articles were further contacted without any further discovery of additional publications related to this review. The inclusion and exclusion criteria were established to ascertain the highest quality studies to answer our clinical question.

Inclusion

- Level I and II evidence studies
- English language studies only
- Human OR animal subjects where tendon repairs have occurred
- Study publication date from 1 January 1950 to current
- Studies investigating treatment of tendon repairs with 5FU

Exclusion

- Level III, IV, V, VI, VII evidence
- Non‐English language studies
- Studies investigating treatment of tendon adhesions with other agents other than 5FU

Tables 1 and 2 identifies all databases and search criteria citation results. Five hundred and forty‐six articles were found over the four databases. The title, abstract, and key words of all articles were then screened for relevance. After exclusion criteria and removal of duplicate studies, 12 studies met inclusion criteria, which were further analysed. Two articles were excluded upon full text evaluation. Cerovac et al^5^ was excluded, as it was assessing tensile strength of repaired flexor tendons treated with 5FU. McGonagle et al^8^ was excluded because it was a non‐randomized experiment looking at the correlation with reducing the coefficient of friction in unrepaired tendons with three synthetic agents including 5FU. Ten articles were then reviewed independently by two authors and data extracted.

**TABLE 1:** Search terms and databases pre‐exclusion criteria

Articles pre‐exclusion criteria	Cochrane	PubMed	MEDLINE	Embase
Tendon adhesions and fluorouracil	1	15	5	7
Tendon adhesions and agents	8	54	4	3
Tendon adhesion and cytotoxic	1	3	1	1
Peritendinous adhesions and agents	0	14	7	2
Tendon adhesions and adjuvants	2	10	1	0
Flexor tendon repair and 5FU	1	13	4	5
Extensor tendon repair and 5FU	0	0	0	0
Tendon injuries and antimetabolites	0	87	5	0
Achilles tendon repair and 5FU	0	2	0	0
Achilles tendon and cytotoxic	0	8	9	20
Tendon adhesions and prevent	30	172	21	30
Total	43	378	57	68

Abbreviation: 5FU, 5‐fluorouracil.

**TABLE 2:** Search terms and databases postexclusion criteria

Articles postexclusion criteria	Cochrane	PubMed	MEDLINE	Embase
Tendon adhesions and fluorouracil	0	11 (4)	3 (2)	4 (3)
Tendon adhesions and agents	0	2 (52)	0 (4)	0 (3)
Tendon adhesion and cytotoxic	0	0 (3)	0 (1)	0 (1)
Peritendinous adhesions and agents	0	1 (13)	0 (7)	0 (2)
Tendon adhesions and adjuvants	0	0 (10)	0 (1)	0
Flexor tendon repair and 5FU	0	10 (0)	1 (0)	2 (3)
Extensor tendon repair and 5FU	0	0	0 (0)	0
Tendon injuries and antimetabolites	0	10 (77)	5 (0)	0
Achilles tendon repair and 5FU	0	0	0 (0)	0
Achilles tendon and cytotoxic	0	0 (8)	0 (9)	0 (20)
Tendon adhesions and prevent	0	2 (170)	0 (21)	0 (30)
Total	0	36 (344)	9 (35)	6 (62)
Total included (excluded)	51 (441)			

Abbreviation: 5FU, 5‐fluorouracil.

An attempt was made to identify common outcome measure(s) across all studies analyzed. However several different clinical outcome measures were used for assessment including inflammatory response, work of flexion, synovial thickening, and tendon load. Studies subject data, treatment dose, significance level, follow‐up, and conclusions were analyzed. A *P* < .05 was a priori deemed statistically significant. The lack of subject‐level specific data and heterogeneity in outcome reporting precluded meta‐analysis.

## 3 RESULTS

**FIGURE 1:**
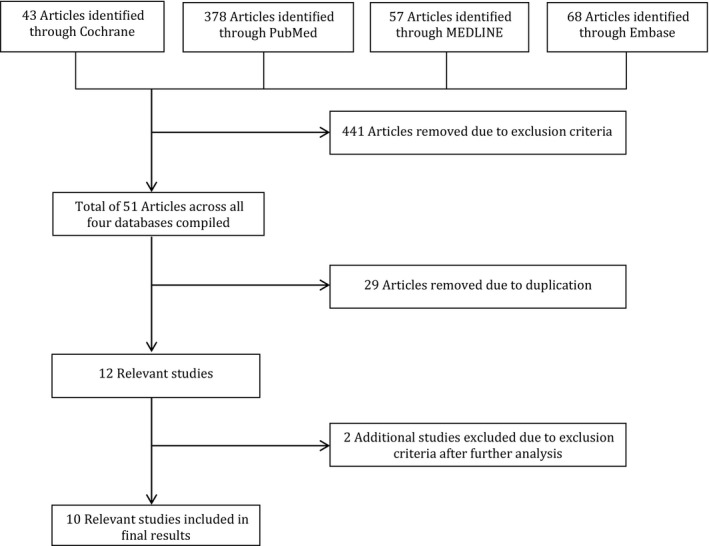
The PRISMA flow diagram illustrating the selection procedure for article inclusion. A total of 546 studies were identified through free text searching of the Cochrane library, Pubmed, Medline and Embase databases. Irrelevant articles, duplications, and articles not published in English were removed, leaving 12 studies for full manuscript review. Of these two did not meet the inclusion criteria, leaving 10 articles

The literature review results are summarized in the PRISMA flow diagram (Figure 1). The authors independently searched key words in the databases to identify 546 publications. Screening of titles, abstracts, and key words highlighted 12 relevant articles for full manuscript review. Reference lists and author contacts of those articles revealed no further studies. Independent review of the 12 studies identified 10 meeting inclusion criteria.

All articles identified were randomized control trials using 5FU in tendon repairs. For discussion, all subjects in the studies were animal studies as there were no human trials at the time of the review. Total cohort size was 267 (130 rabbits, 60 dogs, and 77 chickens). Two studies disclosed financial conflict of interest in part funding their research (Zhao et al^9^; Khan et al^10^). Eight studies reported no other conflict of interest. Follow‐up ranged from 1 day (Khan et al^10^) to up to 42 days postsurgery (Zhao et al^9^). The dosage used ranged from 1 mg/mL to 50 mg/mL of 5FU topically to repaired tendons. Nearly all studies used 5 minute exposures expect for Karaaltin et al^11^ who wrapped slow‐releasing biodegradable gelatin infused‐5‐FU blocks to the repaired tendons. The details of these articles have been summarized in Table 3.

**Table 3:** A summary of the 10 articles and their major findings, subjects, routes of administration, *P* values, and duration of follow‐up

Title and year	Number of subjects	Route of administration	P value	Follow‐up	Major findings
Modulation of the formation of adhesions during the healing of injured tendons Khan et al (2000)^12^	15 rabbits, but 60 tendon operations	5FU single 5 min exposure of 50 mg/mL	<.05	7 d	Inflammatory responses (using level of expression of vascular cell adhesion molecule‐1 as a surrogate) were significantly reduced in the treated tendons
Effects of 5‐fluorouracil on flexor tendon repair Moran et al (2000)^13^	47 chickens	5FU single 5 min exposure of 5, 25, and 50 mg/mL doses	<.05	3 wk	Work of flexion was reduced Decreased adhesion formation in all 5FU‐treated animals
Decrease in adhesion formation by a single application of 5‐fluorouracil after flexor tendon injury Akali et al (1999)^14^	15 rabbits but 30 tendons	5FU single 5 min exposure of 50 mg/mL	<.05	7 d	Significant reduction in synovial sheath thickening (by 30%), cell counts (by 30%), and proportional length of adhesions (by 50%)
Single exposures to 5‐fluorouracil: a possible mode of targeted therapy to reduce contractile scarring in the injured tendon Khan et al (1997)^10^	30 rabbits	5FU single 5 min exposure of 1, 2.5, and 25 mg/mL	<.05	Days 1, 3, and 7 posttreatment	Degree of fibroblast‐populated collagen lattice contraction was significantly inhibited
Biomechanical and macroscopic evaluations of the effects of 5‐fluorouracil on partially divided flexor tendon injuries in rabbits Duci et al (2015)^3^	32 rabbits, 64 tendons	5FU single 5 min exposure of 25 mg/mL	<.05	4 wk	Decrease in adhesion formation in the group treated with 5FU due to increased resistance to tendon adhesions during their excursion through the tendon sheath, which required greater traction force 5FU does not affect the gliding of partially divided flexor tendons that have not been surgically repaired
Histological evaluation of the 5‐fluorouracil on partially divided flexor tendon injuries in rabbits Duci et al (2017)^15^	16 rabbits, 32 tendons	5FU single 5 min exposure of 25 mg/mL	<.05	4 wk	Effective in controlling peritendinous adhesions following surgical repair In partially divided flexor tendons that have not been surgically repaired, 5FU did not affect the creation of adhesions
Surface treatment with 5‐fluorouracil after flexor tendon repair in a canine in Vivo model Zhao et al (2009)^9^	60 dogs (30 treatment, 30 control) 2nd and 5th FDP on treatment tendons zone II	5FU single 5 min exposure of 50 mg/mL	<.05	10, 21, and 42 d	Normalized work of flexion of 5FU treated tendons was significantly lower at 10 d but no difference at 21 or 42 d No significant difference in gliding resistance, repair failure strength, or stiffness at any time point or in histological appearance between groups Expression of type 1 and III collagen and transforming growth factor‐β1 of repaired tendons treated with 5FU was significantly lower at 10 d postoperatively but not at 21 or 42
The effects of 5‐fluorouracil on flexor tendon healing by using a biodegradable gelatin, slow‐releasing system: experimental study in a hen model Karaaltin et al (2012)^11^	30 chickens (3rd and 4th flexor tendons)	10, 20, 30 mg 5FU in gelatin blocks, wrapped around tendons for duration of healing period ie 3 wk	<.05	3 wk	Sustained release of 10 mg loaded gelatin‐loaded group showed a decrease in adhesion formation when compared to control 20‐30 mg groups showed signs of severe inflammation
Prevention of peritendinous adhesion formation after the flexor tendon surgery in rabbits Fatemi et al (2018)^16^	16 rabbits (index and ring fingers of zone II flexor tendon)	5FU single 5 min exposure of 50 mg/mL IFN‐α solution (106 IU/mL), IFN‐β solution (106 IU/mL)	<.05	3 wk	The force and time needed for removing the tendon from its sheath were proportional to the extent of adhesions. The time for removal was significantly shorter in the 5‐FU group Histological examination of the 5FU group showed severe inflammation and adhesions—inconsistent between mechanical and histopathology IFN‐α and IFN‐β did not reduce the peritendinous adhesion formation after the tendon surgery
Reduction in matrix metalloproteinase production by tendon and synovial fibroblasts after a single exposure to 5‐fluorouracil Ragoowansi et al (2001)^17^	8 rabbits (64 flexor digitorum profundus tendons)	5FU single 5 min exposure of 0.25, 0.5, 2.0, 10.0, 15.0, and 25.0 mg/mL	<.05	1, 3, and 7 d	Inhibition of MMP2 and MMP9 production by synovial fibroblasts 5FU may reduce adhesions by limiting the migratory capacity of synovial fibroblasts

IFN, interferon; MMP, matrix metalloproteinase.

## 4 DISCUSSION

Tendon adhesions are a common problem facing surgeons performing flexor and extensor tendon repairs. An estimated 10% of flexor tendon repairs are complicated by adhesion formation.^18^ Tendon adhesions after repair affect functional outcomes for the patient and can delay rehabilitation. There has been extensive research and review of surgical techniques, pharmacological agents, and mobilization protocols to reduce complications post‐tendon repair; however, most studies have been performed in animals with very few human trials.^19^

The focus on 5FU in these studies reflects the desire to modify the activity of fibroblasts, which play a vital role in the formation of adhesions. As an antimetabolite it modifies adhesion formation by inhibiting the proliferation of inflammatory cells and inhibits the invasion of fibroblasts from outside of the tendon, extrinsic fibroblasts.^2^, ^11^, ^13^ Despite the initial necessity of the formation of scar tissue for the physical joining of the injured tendon, the studies in this review did not report that the use of 5FU resulted in a weaker tendon. The reduction of adhesions in the early phase of repair during immobilization with this agent in the studies is promising without compromising repair.

**FIGURE 2:**
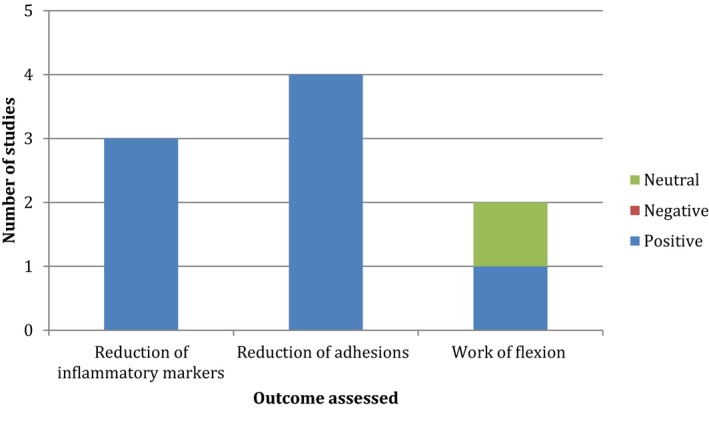
Outcome measures assessed among all studies. Although not all studies were designed with the same primary objective, most of them overlapped in the three main areas listed above. This is a representation of the outcomes measured in the nine positive articles

Nine of the 10 articles concluded that there was benefit to using 5FU to prevent tendon adhesions; however, different focal points in the research were illustrated. The articles came to conclusions in at least one of the three areas: the presence of inflammatory markers, the presence of adhesions, and the strength and load‐bearing capacity of the tendons post‐treatment with 5FU (Figure 2). In all areas 5FU was deemed to be of benefit, bar one article, which saw no benefit, but also no adverse impact.^9^ Three articles described a reduction in the presence of inflammatory markers, two assessed functional resistance and the load required to overcome this, while four assessed the physical presence of adhesions as their core focus. These articles were able to derive a reduction in the presence of adhesions from their research, an outcome that has clear benefits for not only practitioners involved in plastic surgery and orthopaedics but also all those involved in the postsurgery rehabilitation programs. From here it is possible to suggest that the phase 1 clinical application of 5FU as a treatment for the prevention of tendon adhesions.

None of the articles reported any adverse outcomes of the use of 5FU with only one outcome reporting no benefit compared to the control group. Furthermore the authors described an easily replicable application of the drug in comparable doses. This suggests that similar outcomes would be able to be replicated with similar ease. Of note Karaaltin et al^11^ in their 2013 study involving 5FU gelatin‐infused blocks reported significantly higher inflammation at the higher dosing levels of 20 and 30 mg/mL. Of importance however is that this study was the one to use 5FU gelatin‐infused blocks rather than the 5‐minute “single touch” technique adopted by the other nine studies. Finally the outcome of Fatemi et al,^16^ as identified and assessed by its authors found a seemingly contradictory result of histological inflammation and adhesions, but improved mechanical function.

The main limitation of this review is the lack of human studies, with all articles examining either chicken, dog, or rabbit tendons. All studies harvested flexor tendons from their animal subjects. In this manner, the tendons that were used imitated the mechanisms of tendon function and immune responses that would be produced in human studies. This also recognizes the significance of flexor and extensor tendon injury in humans.

Clearly, given the promising nature of the results this could soon be rectified. In a similar light, while two articles reported conflict of interest in light of their funding, eight of the nine other positive articles reported none. Furthermore long‐term follow‐up may be required to assess the longevity of the tendons treated with 5FU as the studies that were assessed focused only on a window of up to 6 weeks without mobilization techniques.

## 5 CONCLUSION

Nine of the 10 articles assessed reported the benefits of 5FU in reducing tendon adhesions postsurgery. Thus, after reviewing the literature and finding congruency across the spectrum of relevant articles it can be asserted that 5FU is of benefit in reducing tendon adhesions and could well indeed find a place among the treatment approaches for reducing tendon adhesions. The results highlight this approach as an avenue that warrants further investigation in the way of human‐focused trials.

## CONFLICT OF INTEREST

None.

## AUTHOR CONTRIBUTION

The main contributors to the article were ON and AS with constant input and guidance from the senior author DN. All the authors have read and approved the final manuscript.
